# Community Readiness for the Promotion of Physical Activity in Older Adults—A Cross-Sectional Comparison of Rural and Urban Communities

**DOI:** 10.3390/ijerph15030453

**Published:** 2018-03-06

**Authors:** Dirk Gansefort, Tilman Brand, Christina Princk, Hajo Zeeb

**Affiliations:** 1 Leibniz Institute for Prevention Research and Epidemiology—BIPS, Achterstr. 30, 28359 Bremen, Germany; brand@leibniz-bips.de (T.B.); princk@leibniz-bips.de (C.P.); zeeb@leibniz-bips.de (H.Z.); 2 Research Focus Health Sciences Bremen, University of Bremen, 28359 Bremen, Germany

**Keywords:** physical activity, older adults, community readiness, primary prevention, capacity building

## Abstract

Communities can play an important role in delivering public health programs to older adults, but they differ in the provision of local structures and resources. The community readiness (CR) approach applies a stage model of change to the community level and analyzes structures and the degree of willingness to take action on a health issue. This study compared the CR regarding the promotion of physical activity as part of healthy ageing for older adults among urban and rural communities in North-West Germany. A cross-sectional CR assessment with key respondents in 23 municipalities (11 urban and 12 rural communities) was conducted using a semi-structured interview. Interviews were scored across the five CR dimensions and global CR score was calculated (scores between 1 = no awareness and 9 = professionalization). Wilcoxon rank-sum test and hierarchical regression models were used to compare urban and rural communities. In total, 118 interviews were conducted (response rate 69.8%). On average, the communities showed moderate CR scores (4.9 ± 0.3; Range: 4.3–5.4; preplanning or preparation phase). The global CR score was slightly higher in rural than in urban communities (regression coefficient = 0.29, 95% confidence interval (CI): −0.02–0.59). The rural communities showed significantly higher CR scores in the ‘Knowledge of efforts’ dimension (0.70, 95% CI: 0.26–1.14) and in the ‘Knowledge of the issue’ (0.37, 95% CI: 0.04–0.70). Rural communities display a slightly higher CR level than urban communities. In the next step, targeted capacity building activities will be initiated among communities with low CR levels.

## 1. Introduction

Physical activity (PA) is an important contributor to healthy ageing [1,2] and it has been shown to be important for reducing the overall burden of disease [3,4]. Community contextual factors have a major influence on PA of community residents. For example, the availability of programs or services, community social and economic conditions, and social networks are associated with better PA behavior [5,6,7,8,9]. Spatial variations and characteristics of the sociocultural environment in communities, for example, the degree of urbanization as well as the varying availability of infrastructures, networks, and other resources, play a major role in shaping the PA profile of communities. For instance, residents of rural communities might have less access to preventive PA promotion services due to more scattered structures, greater geographic dispersion, and more transportation challenges like the absence or poorer availability of public transport or the need to rely on car transport [10]. On the other hand, urban neighborhoods may have lower levels of social cohesion and sense of community. This may lead to a lower attachment to community-based PA clubs or promotion programs [11]. Research from the USA indicates that PA levels in rural communities are lower than in urban and sub-urban communities [12,13]. However, these results may not adequately represent the situation in Western European rural environments. The few studies that investigated this association in the European context provide no clear picture. A study from Belgium found that individuals in urban areas are more physically active than persons living in rural communities [14]. Conversely, a study from Iceland showed no differences between rural and urban older residents [15] and a study from Poland indicated higher levels of leisure-time PA in rural areas [16]. Results from Ireland suggest higher levels of physical inactivity among older women in rural areas but less inactivity among older men in these areas [17].

Among the contextual factors, community readiness (CR) is the degree to which a community is willing and prepared to take action on a specific health issue. The CR concept applies a stage-based behavior change model to the community level [18,19]. It suggests that a certain degree of problem awareness and preplanning in the community is necessary for a health promotion intervention to be implemented successfully [18,19,20]. Thus, it is recommended to assess and, if necessary, increase CR before starting an intervention. Depending on the stage of CR, the model suggests different strategies to enhance program implementation. A major strength of this model is its structured and systematic approach to assess and foster community capacities [21]. The CR model has been used in diverse fields of prevention and health promotion, such as HIV/AIDS, substance use, or obesity prevention. However, its utilization for analyzing community-based PA programs and activities for older adults has not yet been investigated [22,23,24,25,26,27,28]. In this study, we defined older adults as persons aged 65 years and above.

The purpose of this study was to systematically assess the readiness of a community regarding PA promotion for older adults and subsequently enhance community capacities for PA promotion among older adults. Under the assumption that CR is strongly influenced by urban versus rural characteristics and community contexts [29], we focused on analyzing CR differences between urban and rural settings. Our study was conducted in the framework of the regional prevention research network Physical Activity and Health Equity: Primary Prevention for Healthy Aging (AEQUIPA) in North-West Germany [30].

## 2. Materials and Methods

A cross-sectional CR assessment was carried out to determine the level of readiness regarding the promotion of PA for older adults in urban and rural communities.

### 2.1. Design and Sample

The CR assessment was conducted in 23 communities (11 urban, 12 rural) in the Metropolitan Region in the North-West of Germany. Here, the term community refers to political and geographical areas where a group of people live in the same locality and under the same administration. The Metropolitan Region in the North-West of Germany consists of approximately 150 communities. Communities were selected if they had a comparatively high proportion of older residents or if a high increase in the proportion of older adults was expected over the next decade. Population data from local and regional statistics offices were used to identify eligible communities [31].

Interviews with key respondents were conducted from April to August 2015. Key respondents were local stakeholders for the topic of PA among older adults, for example, representatives from local public authorities, senior citizen advocacy groups, sports clubs, for-profit PA providers, and members of the target group (i.e., older adults living in the community). We further identified key respondents via online searches. In addition, we asked respondents for other eligible key respondents in their community. For each community, we aimed to recruit at least one key respondent from public authorities (e.g., mayor, officers for senior and/or social affairs), one from the local sports clubs (e.g., chairperson of the club), one from senior citizen advocacy groups, and one from civil or public services (e.g., community center). Interviews were conducted either face-to-face or by telephone by trained and experienced interviewers. Each interview was digitally recorded, with the participant’s written consent (in the case of face-to-face interviews) or recorded oral consent (in the case of telephone-based interviews). Interviews were professionally transcribed ad verbatim. More details on the methods are provided in the Ready to Change study protocol [32].

### 2.2. Community Readiness Assessment

The interview guide was adapted according to the procedures described in the CR manual [20]. The questions and the scoring instructions were translated into German and then reviewed and revised by a native English-speaking scientist. In a validation process, we tested the comprehensibility of the interview guide in two steps with key respondents from communities that were not part of the study area. After each question, we asked for comprehension and revised some vague wording. The questionnaire contained a set of closed questions (yes/no format) and open-ended questions about the community’s attitudes, knowledge, and beliefs about the issue, addressing the five dimensions of CR [20]:

1. Community efforts and knowledge of efforts,
2. Leadership,
3. Community climate,
4. Community knowledge of the issue, and
5. Resources.

A scoring system was applied to determine the CR [20]. Each interview was scored to provide a readiness level for every dimension using a nine-point rating scale. The inter-rater reliability of independent scoring by two researchers was assessed using the intraclass correlation (ICC), with good to excellent scores ranging between 0.67 and 0.81 [33] (see Table S1). The two scorers discussed each dimension’s score for each interview and jointly decided on a consensual score. The global CR score was mapped to one of the nine stages of CR that are described in Table 1.

Apart from the closed scoring questions, the interview guide provided open-ended questions that we did not analyze in depth for this publication.

### 2.3. Data Analysis

CR scores were calculated for each respondent and then summarized as mean scores at the community level. Consensus mean readiness scores for the five dimensions and a global readiness score per community were calculated, the latter being the arithmetic mean of the five key dimensions. As suggested by Kostadinov et al. [21], the CR scores were presented with standard deviation and range. Characteristics of the key respondents were compared between urban and rural communities using Chi-square tests for categorical variables and Wilcoxon rank-sum test for continuous variables. In the unadjusted analysis of differences in the CR, we compared urban versus rural communities at the cluster (community) level, using the mean score of the communities. Non-parametric tests were applied (Wilcoxon rank-sum test). To adjust the comparison for differences in the respondents’ characteristics (key respondent group, age, self-reported sex, place of residence), we applied a random-effects linear regression model with robust standard errors, using readiness scores at the respondent level. All analyses were performed with Stata 12 (Stata Corp., College Station, TX, USA).

### 2.4. Ethics Statement and Consent

The study protocol was approved by the Ethics Committee of the University of Bremen on 11 February 2015 and published in January 2016 [32] (German Clinical Trials Register DRKS00009564). All participants in the CR interviews received written or oral information about the study. All interviewees gave informed consent for their data to be used. All procedures were in line with the Declaration of Helsinki.

## 3. Results

### 3.1. Sample Characteristics

Of the 196 contacted potential respondents, 51 were unavailable for an interview (due to time restrictions or no cause specified), 27 were not eligible, and 118 were willing to participate. The response rate among the eligible was 69.8%. On average, five (range: 4–8) interviews were conducted in each community. The mean duration of the interviews was 28 min (range: 15–69 min). The age of the respondents ranged from 26 to 78 years (mean = 57; ±13 years), with respondents from rural communities being older than those from urban communities (Table 2). In regard to self-reported sex, 55.1% of the respondents were female. About two-thirds of the respondents were living in the respective community. This proportion was significantly higher in the rural than in the urban communities. Most of the key respondents were representatives from civil and public services (e.g., neighborhood management, community center (*n* = 42) and sports club/facilities (*n* = 38)). In rural communities, we included more representatives from sports clubs and senior advocacy groups; in urban communities, we included more representatives from public services.

### 3.2. Community Readiness

**Figure 1:**
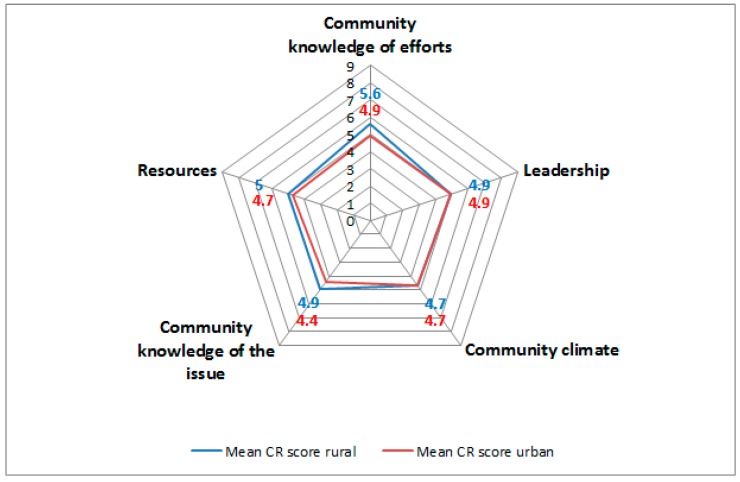
Cobweb chart showing average community readiness (CR) score per dimension for rural and urban communities.

The CR score for all 23 communities was 4.9 (±0.3; 4.3–5.4). This score corresponds to the preplanning phase for the promotion and support of PA activities in older adults (Table 1). The scores for each dimension of the CR assessment are shown in Figure 1. The highest score was seen in the ‘community efforts and knowledge of efforts’ dimension, with 5.3 (±0.6; 4.1–6.5). The lowest score was observed in the ‘community knowledge of the issue’ dimension, with 4.6 (±0.5; 3.5–5.3). Here, only the score for the ‘community efforts and knowledge of efforts’ dimension reached the preparation phase; the other four dimensions were mapped to the preplanning phase. Overall, 15 communities (9 urban and 6 rural) had scores indicative for the preplanning phase and 8 communities (2 urban and 6 rural) for the preparation phase.

### 3.3. Comparison or Rural and Urban Community Readiness

The global CR score on the community level differed between rural and urban communities, with a higher CR score in rural communities (rural = 5.0, urban = 4.7, Wilcoxon rank-sum test, *p* = 0.04). Specifically, rural communities received higher scores in the ‘community efforts and knowledge of efforts’ dimension (rural 5.6, urban 4.9, *p* = 0.01) and the ‘community knowledge of the issue’ dimension (rural 4.9, urban 4.4, *p* = 0.01) (Figure 1).

In the analysis adjusted for key respondent groups, respondent’s age, sex, and place of residence (Table 3), rural communities scored higher in the ‘community efforts and knowledge of efforts’ dimension (+0.70, 95% confidence interval (CI): 0.26–1.14) and in the ‘community knowledge of the issue’ dimension (+0.37, 95% CI: 0.04–0.70). Furthermore, the model showed that female respondents rated the dimension ‘community efforts and knowledge of efforts’ higher and the dimension ‘community leadership’ lower than male respondents. In the ‘community knowledge of the issue’ dimension, responses from public authorities, as well as from senior citizen advocacy groups, indicated a lower readiness than responses from civil and public services (Table 3).

**Figure 2:**
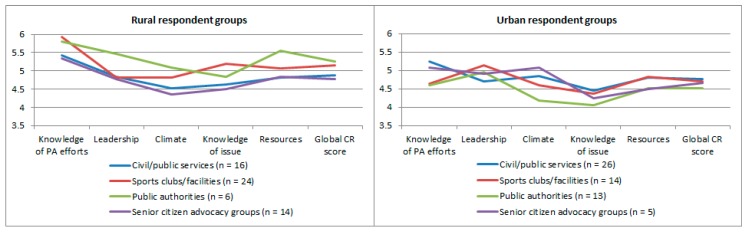
Community readiness scores per dimension and global score, stratified by rural and urban key respondents. PA: physical activity.

We further stratified CR scores by key respondent groups. All rural key respondents groups showed higher means in the dimensions ‘knowledge of PA efforts’, ‘knowledge of the issue’, and ‘resources’. Respondents from rural public authorities showed higher CR scores compared to other rural respondent groups. Figure 2 provides an overview of respondent group stratified results. 

## 4. Discussion

Our study aimed to systematically assess communities’ readiness regarding PA promotion in older adults. In general, communities were found to be at moderate levels of readiness to address PA promotion for older adults, with slightly better readiness scores in rural communities. A total of 15 communities were mapped to the preplanning phase (stage 4) and 8 to the preparation phase (stage 5). Being in the preplanning phase meant that there was recognition amongst at least some community members that physical inactivity might be a local problem and that it should be addressed. There were identifiable leaders, and in some cases there was already a working group addressing this issue. However, efforts to promote PA in older adults were not focused or detailed, and there was no systematic planning of actions to address the problem [19]. Communities mapped to the preparation phase showed more detailed planning. There was more general knowledge and information about local problems with physical inactivity in older adults, although this information was not based on formally collected data but more on word of mouth. Leadership was more active than in the communities that were in the preplanning phase and first decisions were made regarding finding partners and resources for the promotion of PA in older adults.

Although the CR model builds on a long history of application for diverse health issues, our literature searches indicated that it has not yet been used in the European or German context, and rarely for the assessment of CR for PA promotion in older adults. A study from Jones et al. [34] used the CR model to analyze the efforts of communities to promote PA among older adults with arthritis. While other research indicated that rural communities were facing more challenges in implementing and sustaining public health interventions [13], the stage of readiness in this study did not differ between rural and urban communities [34]. Another study from the US assessed the CR model for a community-wide childhood obesity prevention intervention. Here, like in our study, the ‘community efforts and knowledge of efforts’ dimension was the highest, whereas the ‘community climate’ and ‘community knowledge of the issue’ dimensions showed the lowest dimension scores. This similar finding indicates that communities were likely launching and planning efforts, but that the implementation climate was comparably unsupportive.

In our study, the differences between rural and urban communities in the ‘community knowledge of efforts’ and ‘community knowledge of the issue’ dimensions may be due to a higher density of social networks in these rural communities and a stronger sense of belonging to the community, where people were aware about the efforts that were being made related to the issue [11,35]. On the other hand, there was a broader spectrum of different efforts in urban communities. Respondents from urban communities reported on average about five different PA activities for older adults, whereas respondents from rural communities reported three different activities. The higher complexity and diversity may be a reason for the lower level of overall knowledge about local efforts, as an overview may be more difficult to attain. Here, the high level of community knowledge in urban communities was a strength and could be used to mobilize stakeholders. For urban communities, more efforts to increase knowledge and awareness may be necessary. A provision of accounts of the diverse community activities and sport facilities can be useful. Generalization of our findings to other countries or regions is difficult because in countries with a greater geographic dispersion (like the US, Canada, or Australia) the challenges in reaching PA facilities are higher than in our case. Factors of the communities’ built environments, such as the availability of a public transportation system and a walk-friendly infrastructure, may play roles as central resources for the promotion of PA [36]. In addition, community readiness relies on the availability and functioning of public authorities and/or stakeholders from the civil society for the formation of a community coalition. Although we covered communities with different degrees of deprivation in our sample, we could approach an existing set of community stakeholders in all communities. In very deprived areas, however, the formation of a community coalition may be more difficult.

The response rate of nearly 70% indicated a high motivation and acceptability by local key respondents to take part in the assessment and discuss PA for older adults in their community. With an average age of respondents of 57 years, we were able to include individuals from our target group of older adults as well as stakeholders from the targeted institutions like sports clubs, local administration, and civil services. Thus, a broad spectrum of views and attitudes from the community was obtained and the identified key respondents were quite knowledgeable about the issue.

We identified some limitations while working with the assessment instrument. Response bias may have occurred due to the fact that key respondents provided answers from their own personal points of view. These answers might not always have given an accurate representation of their community’s readiness. On average, we conducted five interviews per community, as recommended by the developers of the instrument, however, this may not be enough to obtain a full picture of the CR in a community. Further validation thus appears necessary to assess the stability of CR scores, for example, through conducting a larger number of interviews per community. Further, our study did not link CR with individual data from community residents, which would have given a better overview of the level of PA in older adults and the connection to community contextual factors.

Apart from assigning the communities to a certain readiness stage, the assessment showed that every community has its own individual characteristics. The qualitative information gained from the interviews enhanced the study team’s understanding of the communities. The assessment provided useful information on the communities’ profiles for planning practical strategies to increase community capacities. Therefore, the next step in our project was to build capacities for PA promotion in selected communities, with the aim to increase the CR to the next level, (i.e., to the initiation (stage 6) or stabilization (stage 7) phase, respectively). To increase the capacity of communities, we used strategies that included information about physical inactivity in older adults and efforts to promote PA in the local media and via public information forums. Further, we installed local working groups to discuss the issue and to take action on it. After these measures, a community-based PA promotion intervention for older adults will be provided [37]. After a three-year interval, we will perform a follow-up CR assessment to analyze the effectiveness of these capacity-building efforts.

## 5. Conclusions

This study illustrated that the CR model is applicable for the health issue of physical inactivity in older adults. Communities showed moderate CR scores and the differences between rural and urban communities suggested an adjusted procedure in capacity building regarding the contextual factors of a community. To conclude, the community readiness model is a promising and useful theory-based strategy to analyze communities’ contextual structures. Further, interviews with key respondents might be a first step to raise the awareness of a community about a health issue like physical inactivity in older adults.

## Figures and Tables

**Table 1:** Stages of community readiness.

Stage	Title	Description
1	No awareness	Issue is not generally recognized by the community or leaders as a problem (or it may truly not be an issue).
2	Denial/resistance	At least some community members recognize that it is a concern, but there is little recognition that it might be occurring locally.
3	Vague awareness	Most feel that there is a local concern, but there is no immediate motivation to do anything about it.
4	Preplanning	There is clear recognition that something must be done, and there may even be a group addressing it. However, efforts are not focused or detailed.
5	Preparation	Active leaders begin planning in earnest. Community offers modest support of efforts.
6	Initiation	Enough information is available to justify efforts. Activities are underway.
7	Stabilization	Activities are supported by administrators or community decision makers. Staff are trained and experienced.
8	Confirmation/expansion	Efforts are in place. Community members feel comfortable using services, and they support expansions. Local data are regularly obtained.
9	Community ownership/Professionalization	Detailed and sophisticated knowledge exists about prevalence, causes, and consequences. Effective evaluation guides new directions. Model is applied to other issues.

**Table 2:** Characteristics of the key respondents.

Key Respondents	Overall (*n* = 118)	Rural Communities (*n* = 60)	Urban Communities (*n* = 58)	*p*-Value *
Self-reported sex (% male/female)	44.9/55.1	45.0/55.0	44.8/55.2	n.s.
Age in years (mean (±))	57.0 (±12.6)	60.7 (±11.1)	53.1 (±12.9)	0.002
Living in respective community (% Yes (*n*))	67.8 (117)	88.3 (53)	46.6 (27)	0.000
Representative from:				
Civil and public services % (*n*)	35.6 (42)	26.7 (16)	44.8 (26)	0.008
Sports clubs/facilities % (*n*)	32.2 (38)	40.0 (24)	24.1 (14)	
Public authorities % (*n*)	16.1 (19)	10.0 (6)	22.4 (13)	
Senior citizen advocacy groups % (*n*)	16.1 (19)	23.3 (14)	8.6 (5)	

Statistical tests: Chi square test for categorical variable; Wilcoxon rank-sum test for continuous variables; * *p* < 0.05; n.s.: not significant.

**Table 3:** Comparison of community readiness scores of rural versus urban communities.

	Global Community Readiness Score	Community Knowledge of Efforts	Community Leadership	Community Climate	Community Knowledge of the Issue	Community Resources
	RC (95% CI)	RC (95% CI)	RC (95% CI)	RC (95% CI)	RC (95% CI)	RC (95% CI)
**Geographical Area**						
Urban	Ref.	Ref.	Ref.	Ref.	Ref.	Ref.
Rural	0.29 (−0.02, 0.59)	0.70 (0.26, 1.14) *	0.11 (−0.35, 0.58)	−0.09 (−0.47, 0.29)	0.37 (0.04, 0.70) *	0.35 (−0.11, 0.82)
**Key Respondent Groups**						
Civil and public services	Ref.	Ref.	Ref.	Ref.	Ref.	Ref.
Sports clubs/facilities	−0.04 (−0.32, 0.24)	−0.04 (−0.42, 0.23)	−0.05 (−0.53, 0.42)	0.09 (−0.32, 0.50)	−0.16 (−0.60, 0.28)	−0.05 (−0.40, 0.29)
Public authorities	−0.31 (−0.65, 0.03)	−0.23 (−0.79, 0.32)	−0.15 (−0.48, 0.18)	−0.30 (−0.85, 0.25)	−0.57 (−1.05, −0.08) *	−0.27 (−0.67, 0.13)
Senior citizen advocacy groups	−0.11 (−0.32, 0.10)	−0.14 (−0.53, 0.26)	−0.10 (−0.52, 0.72)	−0.24 (−0.83, 0.34)	−0.47 (−0.80, −0.16) *	−0.07 (−0.55, 0.40)
**Respondent’s Age**	0.00 (−0.01, 0.01)	0.01 (−0.01, 0.02)	0.00 (−0.01, 0.01)	0.00 (−0.01, 0.01)	0.01 (−0.01, 0.02)	0.00 (−0.02, 0.01)
**Self-Reported Sex**						
Male	Ref.	Ref.	Ref.	Ref.	Ref.	Ref.
Female	0.00 (−0.01, 0.01)	0.47 (0.11, 0.83) *	−0.35 (−0.68, −0.02) *	−0.12 (−0.44, 0.20)	−0.19 (−0.50, 0.11)	−0.27 (−0.55, 0.01)
**Living in Respective Community**						
Yes	Ref.	Ref.	Ref.	Ref.	Ref.	Ref.
No	−0.03 (−0.28, 0.23)	0.10 (−0.40, 0.59)	0.17 (−0.14, 0.49)	−0.36 (−0.89, 0.17)	−0.17 (−0.47, −0.14)	0.08 (−0.45, 0.61)

Random-effects generalized least square regressions with robust standard errors, adjusted for community cluster effects; number of observation = 118, number of clusters = 23, average cluster size = 5.1 (range 4–8); RC regression coefficient; CI confidence interval; * *p* < 0.05.

## Supplementary Materials

The following are available online at http://www.mdpi.com/1660-4601/15/3/453/s1, Table S1: Community readiness scores per dimension and rural/urban communities and intraclass correlation (ICC).
