# Supplementary material for: Community Readiness for the Promotion of Physical Activity in Older Adults—A Cross-Sectional Comparison of Rural and Urban Communities
Source: Int J Environ Res Public Health. 2018 Mar 6;15(3):453. doi: 10.3390/ijerph15030453 (PMC5876998; doi:10.3390/ijerph15030453)
Supplement: Supplementary file 1 [file ijerph-15-00453-s001.docx]

**Table S1.** Community readiness scores per dimension and rural/urban communities and intraclass correlation (ICC).

| **Urban/Rural Community** | **Global CR Score** | **Community Knowledge of Efforts** | **Leadership** | **Community Climate** | **Community Knowledge of the Issue** | **Resources** | **Number of Interviews** |
| --- | --- | --- | --- | --- | --- | --- | --- |
| Overall  mean (±; range) | 4.86  (±.30; 4.33–5.41) | 5.30  (±.63; 4.05–6.5) | 4.86  (±.46; 3.94–5.6) | 4.66  (±.39; 3.75–5.38) | 4.61  (±.46; 3.45–5.33) | 4.84  (±.48; 3.83–5.71) | 118 |
| Rural  mean (±; range) | 4.99  (±.30; 4.48–5.41) | 5.63  (±.53; 4,75–6.5) | 4.87  (±.55; 3.94–5.6) | 4.66  (±.32; 4.2–5.3) | 4,85  (±.36; 4.19–5.33) | 4,97  (±.41; 4.31–5.71) | 60 |
| Urban  mean (±; range) | 4.71  (±.23; 4.33–5.03) | 4.94  (±.54; 4.05–5.69) | 4.86  (±.37; 4.2–5.5) | 4.65  (±.46; 3.75–5.38) | 4.35  (±.44; 3.45–4.92) | 4.70  (±.52; 3.83–5.38) | 58 |
| ICC | 0.81 | 0.67 | 0.74 | 0.71 | 0.77 | 0.78 | - |
